# Seroprevalence of Markers of Hepatitis B Virus Infection, Associated Factors, and Vaccination Status in Young Adults in Arkhangelsk, Northwest Russia: A Population-Based Cross-Sectional Study

**DOI:** 10.3390/ijerph15091905

**Published:** 2018-09-01

**Authors:** Tatiana Balaeva, Andrej M. Grjibovski, Olga Samodova, Anatoly Sannikov, Elise Klouman

**Affiliations:** 1 Department of Community Medicine, UiT The Arctic University of Norway, 9037 Tromsø, Norway; elise.klouman@gmail.com; 2 Institute of Public Health, Northern State Medical University, 163000 Arkhangelsk, Russia; jsannikov@yandex.ru; 3 Center of Hygiene and Epidemiology in the Arkhangelsk Region, 163001 Arkhangelsk, Russia; 4 Central Scientific Research Laboratory, Northern State Medical University, 163000 Arkhangelsk, Russia; andrej.grjibovski@gmail.com; 5 Department of Public Health, Health Care, General Hygiene and Bioethics, North-Eastern Federal University, 677000 Yakutsk, Russia; 6 Department of Health Policy and Management, Al-Farabi Kazakh National University, 050038 Almaty, Kazakhstan; 7 West Kazakhstan Marat Ospanov State Medical University, 030010 Aktobe, Kazakhstan; 8 Department of Infectious Diseases, Northern State Medical University, 163000 Arkhangelsk, Russia; ovsamodova@mail.ru

**Keywords:** population-based study, hepatitis B prevalence, hepatitis B vaccination, Russia

## Abstract

Russia had a high incidence of hepatitis B virus (HBV) infection before the vaccination campaigns of 1997, 2001, 2007, which targeted newborns, adolescents, and adults, respectively. The aim of our study was to assess the prevalence of serological markers of HBV infection, associated factors, and vaccination status among young adults in Arkhangelsk, Northwest Russia. In this cross-sectional, population-based study, we used a quota sampling method to recruit 1243 adults aged 18–39 years. Participants completed a self-administrated questionnaire and were tested for hepatitis B markers. Associations between positivity for markers and selected sociodemographic and behavioral factors were studied by logistic regression. 10.9% of our participants were positive for at least one marker of hepatitis B, 1.2% were positive for HBsAg, and 42.1% were negative for all markers. In multivariable logistic regression analyses, age 30–34 years; lack of self-reported vaccination; and having ≥2 sexual partners in the last 6 months were associated with positivity for markers of hepatitis B. Hepatitis B vaccination was confirmed in 46.9% of participants. Although half of our study sample was vaccinated, four in 10 were still susceptible to infection and more than one participant in 100 showed evidence of an active infection.

## 1. Introduction

Infection with hepatitis B virus (HBV) is a major public health problem that is associated with high morbidity, high mortality, and high treatment-related costs. Approximately one-third of the global population has serological markers of HBV infection, and more than 240 million people suffer from chronic hepatitis B [1]. About 800,000 people die each year from complications related to HBV infection, such as cirrhosis and liver cancer [2]. HBV can be transmitted through percutaneous or mucosal exposure to infected blood or other body fluids [3]. It can be transmitted through sexual contact, both heterosexual and in men who have sex with men; persons with multiple sexual partners; from mother to child during pregnancy or at birth; among intravenous drug users (IDU); or in healthcare settings where needles are reused [4]. The prevalence of HBV infection is highest among adults aged 19–49 years in sub-Saharan Africa and East Asia (>5%). In post-soviet countries and the Middle East, the prevalence of HBV infection varies between 2% and 4%, while in North America and Western Europe the prevalence of HBV infection is below 2% [5].

In Russia, the history of acute HBV infection in the post-Soviet era can be divided into two periods. The first period was characterized by a substantial increase in the incidence of HBV infection from 22.3 per 100,000 in 1993 to 43.8 per 100,000 in 1999. Since 2000, the incidence of acute HBV infection began to decrease due to the introduction of HBV vaccination, which was first implemented in the Russian Federation in 1990, but it was only administered to high-risk groups in a few regions [6]. Vaccination of newborns was included in the National Immunization Program in 1997, and vaccination of 13-year-olds in 2001. Despite these measures, the incidence of acute HBV infection in Russia remained high (10.0 per 100,000 in 2004). So, in 2007, the vaccination of the adult population was implemented as a part of the national project “Health” [7]. Since then, more than 70 million people, approximately 50% of the total population, have been vaccinated, and the incidence of acute HBV infection decreased to 1.1 per 100,000 in 2015. Registration of chronic HBV infection was initiated in 1999; in 2001 the incidence of chronic HBV infection was 16.0 per 100,000 and by 2015, this number had decreased to 10.8 per 100,000 [8]. According to official statistics, the incidence of both acute and chronic forms of HBV infection in the country is the highest in the age group 20–39 years [8].

In May 2016, the World Health Organization (WHO) formulated the first Global Health Sector Strategy on Viral Hepatitis, with the stated aim to eliminate these infections. Ten core indicators were proposed to control the spread of these infections, such as prevalence of chronic HBV infection, the number of people living with HBV infection, and coverage of a third dose of HBV vaccine among infants [9]. This strategy has two targets that are to be achieved by 2030: reduction of the incidence of chronic HBV infection by 90% and reduction of mortality from HBV infection by 65%. The WHO Regional Office for Europe and the European Centre for Disease Prevention and Control assessed the availability of data for each of the core indicators and concluded that they are insufficient to assess the epidemiological burden in many countries in the European region. Therefore they recommended seroprevalence surveys to monitor the prevalence of viral hepatitis, including undiagnosed infections [10].

The data on serological markers of HBV infection and associated factors in Russia are scarce; we identified only one study in the international peer-reviewed literature [11]. However, the sample in that study consisted of blood donors and patients from outpatient clinics, with questionable external validity for the general population. The Arkhangelsk Region is situated in Northwest Russia and has an incidence of chronic HBV infection that is similar to the average incidence in Russia as a whole [12]. 

To support preventive efforts against HBV, the aim of our study was to assess the prevalence of serological markers of HBV infection, associated factors, and vaccination status among young adults in Arkhangelsk City, Northwest Russia. 

## 2. Materials and Methods

### 2.1. Study Population and Study Design

This population-based cross-sectional study was a part of a large Russian-Norwegian project [13]. The study was carried out in Arkhangelsk City, which has a population of about 350,000. Census data were not available for research purposes, thus the study sample was drawn from the general population using a quota sampling method [14]. The Agency on Social Research in Arkhangelsk has a database of all cellphone numbers in the city, as well as population-based group-level census data on sex and age distribution (“quotas”) in the eight districts of Arkhangelsk City. Using this information, they phoned eligible persons aged 18–39 years and informed them about the aims of the study using a script provided by the study team. Those who agreed to participate presented themselves at the University Clinic of the Northern State Medical University (NSMU) of Arkhangelsk to complete a self-administered questionnaire and provide a blood sample. The enrollment procedure was described previously in detail [15]. Data collection lasted from the end of September 2010 until June 2011. Altogether, 4872 respondents were contacted to fulfil the “quotas” according to gender and age group. Of them, 3973 agreed to participate; however, the study team ended enrollment after 1265 participants, since this sample size was sufficient according to power calculations. Twenty-two participants had missing blood test results and were excluded from the analyses, resulting in a study sample of 1243 participants.

### 2.2. Description of the Variables

The questionnaire collected information on sociodemographic and socioeconomic factors, sexual and preventive behavior, alcohol use, history of sexually transmitted infections (STIs) other than HIV, and history of HIV infection. Participants reported their family income on a scale of 1 to 10, based on how they thought their income compared to the average income in Arkhangelsk City. We then categorized these responses as low (1–4), medium (5–7), and high (8–10) income. The description of most variables can be found elsewhere [15]. 

Self-reported HBV vaccination status was divided into “finished vaccination” (three doses of vaccine), “unfinished vaccination” (one or two doses of vaccine), “unvaccinated” (no dose) and “unknown vaccination status”. Previous blood transfusions, surgery, and ever being a blood donor were collapsed into one variable and divided into the categories: “no medical procedure”, “one of these medical procedures”, “two of these medical procedures”, and “all three of these medical procedures”. Having tattoos was dichotomized into “yes” and “no”. Respondents were asked whether they knew anyone with HBV infection, and responses were divided into “no”, “yes”, and “don’t know”. 

### 2.3. Serological Testing 

Specimens were sent daily to the research laboratory of the NSMU, where they were centrifuged and frozen at −20 °C. Each month, collected serum specimens were transported to the laboratory of the Regional STI Clinic in Arkhangelsk City, where they were tested for HBV Antigen (HBsAg), HBV core antibodies (anti-HBc), HBV surface antibodies (anti-HBs) and Hepatitis C IgG+IgM antibodies, using an enzyme-linked immunosorbent assay. 

Blood samples were also tested for herpes simplex virus type 2 by an enzyme-linked immunosorbent assay for IgG antibodies against the specific HSV-2 glycoprotein g2 [15]. Main results on the hepatitis C testing are reported elsewhere. All assays were performed according to the manufacturer’s instructions. All reagents were manufactured by Vector-Best Company (Novosibirsk, Russia). For the logistic regression analysis participants negative for all serological markers were considered unvaccinated and never-infected, coded as 0. Participants positive for anti-HBs only were considered vaccinated, coded as 0. All other combinations of positivity for serological markers indicated current or previous HBV infection, and were coded as 1 (Table 1). 

### 2.4. Statistical Analyses 

A sample size of 1042 was required to detect odds ratios (ORs) of two or greater in multiple logistic regression analysis with up to seven independent variables for the prevalence of the outcome of 5% or higher with a 95% confidence level. Therefore, we aimed to recruit 1200 subjects, taking into account potential drop-outs and missing data. Categorical variables were analyzed using Pearson’s chi-squared tests. Crude and adjusted logistic regression analyses were conducted to assess the association between positivity for serological markers of HBV infection and potential related factors. All variables that were significantly associated with positivity for serological markers of HBV or had a *p*-value < 0.15 in crude regression analyses were included in the multivariable model, which was used to estimate adjusted ORs and 95% confidence intervals (CIs). All analyses were performed using Statistical Package for Social Sciences version 20 (SPSS Inc., Chicago, IL, USA). Like other authors, we didn’t exclude participants who were positive for anti-HBs only (considered as vaccinated) from analyses of risk factors [16,17,18]. There were two reasons for that. Mass vaccination of the adult population started in 2007, 3 years before our study period. Thus, vaccinated persons were protected only a few years prior to their participation in the study. Furthermore, there was no screening for HBV infection before the HBV vaccination program took effect. 

### 2.5. Ethical Approval

The study was conducted in accordance with the Helsinki Declaration and approved by the Ethical Committee at Northern State Medical University in Arkhangelsk on 15 March 2010 (No. 04/03). The participants were given an invitation letter with information about study aims and procedures, and participants were contacted by telephone if they wanted to know their test results. All participants signed a written informed consent form. Since this was not a clinical, but solely a seroepidemilogical study, participants were also asked to provide an e-mail address or telephone number by which they could be contacted and informed in case tests revealed former or present HBV infection. After learning their laboratory results, 42 participants were referred to a specialist in infectious diseases, free of charge. 

## 3. Results

The study population consisted of 1243 participants, 543 men and 700 women (43.7% and 56.3%, respectively). Most participants had higher or incomplete higher education (58.9%) and 72.0% belonged to the medium income group (Table 2). 

The prevalence of serological markers of HBV infection in the full sample was 10.9%, the prevalence among men was 11.8% and among women 10.2% (Table 1). Among our study sample, 46.4% of men and 38.9% of women were negative for all serological markers, 46.9% were positive for anti-HBs only, indicating that they were vaccinated, 1.2% were positive for HBsAg, and 10.7% were positive for anti-HBc (Table 1). The characteristics of the participants positive for HBs Ag are presented in Table 3. Three of the 15 HBAg positive cases had a co-infection with hepatitis C.

In crude logistic regression analyses, positivity for serological markers of HBV infection was positively associated with older age, low income, ever smoking, self-reported STI (other than HIV), reporting ≥2 partners in the last 6 months, and absence of self-reported HBV vaccination. Higher education was negatively associated with positivity for serological markers of HBV infection, indicating a protective effect (Table 2 and Table 4).

In addition, in crude logistic analyses, several well-known behavioral risk factors were positively associated with positivity for serological markers of HBV infection, although these associations were not significant, i.e., intravenous drug use and sex with intravenous drug users; early age at sexual debut; sex with a partner of the same gender; not using condoms; two or more previous medical procedures; having tattoos; knowing someone with HBV infection; and not considering HBV vaccination necessary. The prevalence of serological markers of HBV infection in these groups was higher compared to their counterparts without these factors. Among the 543 men in our study, 18 (3.3%) reported having sex with a partner of the same gender. In this group, the prevalence of serological markers of HBV infection was 16.7% (95% CI 5.84–39.23), and the crude OR for being positive for serological markers of HBV infection was 1.5 (95% CI 0.42–5.36). The prevalence among men having sex with men was the highest prevalence found in this study, together with those who reported three medical procedures. This group counted also only 18 persons, 16.7% were positive for serological markers for HBV infection, and crude OR was 1.64 (95% CI 0.46–5.82) The prevalence of serological markers of HBV infection among men who had never had sex with men was 11.7% (95% CI 9.23–14.76). Among women, ever having sex with a partner of the same gender was not associated with positivity for serological marker of HBV infection in crude log regression analyses (OR = 1.02; 95% CI 0.30–3.48). 

In our study sample, 42.5% of participants reported that they had received at least one dose of HBV vaccine, and 30.6% did not know whether or not they were vaccinated; however, in the latter group, 43.2% had isolated anti-HBs antibodies. Serological markers of HBV infection were found among 8.4% of men and 7.9% of women who reported receiving three doses of HBV vaccine (Table 2), and 36% and 26%, respectively, did not know if they were vaccinated against HBV. At the same time, the vast majority of participants (>90%) agreed or tended to agree that vaccination against HBV was necessary, and serological testing actually showed that 46.9% were vaccinated. 

In the multivariable logistic regression analysis, age 30–34 years, absence of self-reported vaccination, and having ≥2 sexual partners in the last 6 months were significantly associated with positivity for serological markers of HBV infection, and self-reported history of STI was borderline significant (Table 5).

## 4. Discussion

To our knowledge, our study is the most recent serosurvey on markers of HBV in the general population of Northwest Russia. It was conducted after the initiation of mass HBV vaccination programs in the young adult population, and it combined serological markers of HBV infection and vaccination with data on risk behavior. 

### 4.1. Prevalence of Serological Markers of Hepatitis B Virus Infection and Vaccination

Fifteen persons in our study sample (six men and nine women, 1.2% of the study sample) tested positive for HBsAg and could thus infect others. According to World Health Organization classifications, a prevalence of 1.2% is categorized as a low-prevalence population [3]. Indeed, the prevalence we observed is comparable with that found in the general population of Spain, Italy, and Greece. The same prevalence was found in the Moscow region in 2008 among first-time blood donors and people attending outpatient clinics [11,19]. However, such comparisons should be done with caution, because donors do not always represent the general population and may have a lower prevalence of HBsAg [20]. According to the regional Federal Service for Surveillance on Consumer Rights Protection and Human Wellbeing in Arkhangelsk, the prevalence of HBsAg among blood donors in the region in 2010–2015 was less than 0.2% [21]; however the prevalence of HBsAg is several times higher among young adults in Arkhangelsk City in our study. 

In the present study, positivity for anti-HBc and/or HBsAg combined with negativity for HBsAg was considered as “ever being infected” with HBV. The childhood or youth of our participants coincided with the early post-soviet era. During this period, risk factors for HBV increased in the population, such as intravenous drug use and a freer sexual lifestyle [22,23]. The situation was aggravated by the absence of HBV vaccination. The prevalence of anti-HBc was 10.7% in our study. This figure is lower than that reported from six regions of Russia in 2008 [11]. Our results also fit with the incidence rate of acute HBV infection in the Arkhangelsk Region, which has decreased more during last 20 years than in other regions [11,24]. The prevalence of anti-HBc among our participants was close to that reported in Poland, where 10.3% of adults were positive for this marker [25]. Data from Tajikistan showed that 30% of 15–24-year-olds are positive for anti-HBc, compared to 47% of adults aged 20–59 years in China, and 57.4% of adults aged 20–39 years in Nigeria [16,26,27]. Data from other parts of the world demonstrate that the higher the proportion of the vaccinated population, the lower the proportion of HBV-infected individuals: in Poland 55% of the adult population is vaccinated, and in China and Nigeria these values are 23.7% and 7.9%, respectively [16,25,27]. 

Medical records of our participants were not available for this study, so we asked participants to self-report their vaccination status. Among them, 42.5% reported having at least one dose of HBV vaccine (Table 3), but 46.9% were positive for anti-HBs, only, according to laboratory tests (Table 1). These data correspond pretty well with official statistics, which state that 50% of the adult population in Arkhangelsk was vaccinated by the beginning of 2011 [28]. The impact of universal vaccination in Russia was assessed by the researchers from Moscow. According to their estimations, 91–95% of cases of acute and chronic HBV infection that would otherwise occur are, in fact, prevented by HBV vaccination [11]. Interestingly, 30.6% of our participants didn’t know whether or not they had been vaccinated against HBV. By comparison, in a Chinese population-based survey only 9.2% of adults didn’t know their vaccination status. These data demonstrate another specific feature of Russian society formulated by Russian sociologists: nowadays young adults in Russia have a relatively low level of healthcare seeking and a low level of knowledge about their health [29]. Among young adults in our study, 42.1% were still susceptible to HBV infection, so the mass vaccination campaign for adults should be continued. 

### 4.2. Factors Associated with Positivity for Serological Markers of Hepatitis B Virus

The prevalence of serological markers of HBV among participants aged 30–39 years was two times higher than the prevalence in those aged 18–29 years. The same trend was observed for anti-HBc in the Moscow region [11] and for HBsAg among pregnant women in Bulgaria [30], but it was not observed in surveys from Nigeria, China, India, and the Lao People’s Democratic Republic [16,31,32,33].

In our study, participants with low education or income level had a higher prevalence of serological markers of HBV infection. These markers were negatively associated with higher education and income variables in crude log regression analyses, indicating a protective effect. In multivariable analyses these associations were still negative, but they were not significant. Other studies have also found a higher prevalence of markers of HBV infection among people with the lowest level of education [16,18,34] or income [32].

HBV is highly contagious and easily transmitted sexually [5]. In our study participants with a history of STI and multiple sexual partners (≥2 in the past 6 months) had a higher risk of HBV infection. For the last 7 years sexual transmission of HBV infection has also been one of the most common routes of transmission reported in the Arkhangelsk Region [21]. Multiple sexual partners and a history of STI have been also associated with HBV infection in previous studies [35,36,37]. Men who have sex with men are considered a high-risk group [4]. In our study, positivity for serological markers of HBV was positively associated with belonging to this group, but this association was not significant in crude regression analyses. This can probably be explained by the small number of individuals in this risk group. Indeed, the prevalence of markers of HBV infection among men who have sex with men in our study was close to that observed in the USA (17% versus 19%) [38]. 

Intravenous drug use is an important risk factor for HBV infection, which is also the case in Arkhangelsk region [4,21]. In crude logistic regression injecting drug use was associated with seropositivity of HBV markers (Table 4). However this association was not significant. This might be due to lack of power and insufficient number of participants recognized themselves as ever being drug users.

### 4.3. Strengths and Weaknesses of the Study

Due to the cross-sectional design, a temporal relationship between risk behavior and positivity for markers of HBV could not be established. Moreover, as we investigated the young adult population, so we cannot generalize our results to neither the whole Russian population nor the entire adult population of Arkhangelsk City. We asked if the participants knew any with HBV infection, but we did not ask if family members had present or former HBV infection. Thus, we did not attempt to assess any HBV transmission from mother to child that might have happened nearly 2–4 decades ago. This may be considered as limitation of the study. In addition, the study questionnaire contained sensitive questions, which may have caused social desirability bias due to underreporting of high-risk behavior [39]. Blood sampling can be seen as unpleasant by some, and may have been a deterrent to study participation [40]. Our sampling method cannot rule out selection bias, for instance through recruitment via cellphone owners; therefore generalization of our study results should be avoided. Participants received compensation high enough to pay for a taxi to and from the clinic, and this likely secured participation among people with low income.

The main strength of our study is its population-based design, including both sexes. We chose to study young adults, as this group has the highest risk of getting the infection [8]. Moreover, the most frequent routes of HBV transmission in the region are sexual transmission, intravenous drug use, beauty treatments (e.g., tattoos, pedicures, etc.), and from mother to child, and thus relate to this age group [21]. Another benefit of our study is the time period in which we carried out of the investigation—soon after the completion of mass immunization project in the adult population. Most of the vaccinated adults in Russia were immunized in 2007–2009. So, in 2010–2011 we had a unique chance to investigate herd immunity and the association of risk factors for HBV markers in the general population. Most of our participants were vaccinated only 1–2 years prior to the study; before that they were not protected and were susceptible to HBV infection. According to the official data, the percentage of vaccinated adults aged 18–35 years was more than 80% in 2016 [41]. So, now the study of the associations between risk factors and positivity for HBV markers would be difficult. Another strength of our study is it was designed as a “second-generation HIV/STI survey”, which combines biological indicators with data on risk behavior to provide information for HIV/STI preventive efforts [42] and provide useful data for monitoring the situation according to Global Health Sector Strategy on viral hepatitis [9].

## 5. Conclusions

The prevalence of HBV markers in our study was close to that observed in Southern and Eastern European countries. Although half of our participants were protected against HBV thanks to mass adult vaccination, more than four in ten were still susceptible to HBV infection and there were individuals with active HBV infection of whom most did not know that they were infected. Therefore the work of HBV prevention should be continued at all levels, including federal financial support of free-of-charge HBV vaccination as part of the National Project “Health”, promotion of the HBV vaccination campaign at the regional level, and routine work at the local level, i.e., by recommending that physicians test for and treat HBV infections and encourage their patients to be vaccinated during regular check-ups and before operations; by using mass media and social networks to spread information about HBV infection; and by encouraging people to be vaccinated to protect themselves, their partners, and their children.

## Figures and Tables

**Table 1:** Prevalence of serological markers of HBV infection and HBV vaccination status in a population-based study of adults aged 18–39 years, Arkhangelsk City, Russia, 2010–2011.

Serological Markers of HBV Infection ^a^/Vaccination			Interpretation of Lab Results	Males		Females		Total	
HBsAg	Anti-HBc	Anti-HBs		*n*	%	*n*	%	*n*	%
Negative	Negative	Negative	Uninfected, HBV infection susceptible	252	46.4	272	38.9	524	42.1
Negative	Negative	Positive	Vaccinated	227	41.8	356	50.9	583	46.9
Negative	Positive	Positive	Current or previous HBV infection	31	5.7	53	7.6	84	6.7
Negative	Positive	Negative		27	5.0	10	1.4	37	3.0
Positive	Positive	Negative		5	0.9	1	0.15	6	0.5
Positive	Positive	Positive		1	0.2	5	0.7	6	0.5
Positive	Negative	Negative		0	0.0	2	0.3	2	0.2
Positive	Negative	Positive		0	0.0	1	0.15	1	0.1
Total				543	100	700	100	1243	100

^a^ Serological markers of HBV infection: HBsAg and/or anti-HBc; Abbreviations: HBV: hepatitis B virus; HBsAg: HBV surface Antigen; anti-HBc: HBV core antibodies; anti-HBs: HBV surface antibodies.

**Table 2:** Prevalence of serological markers of HBV infection and crude associations between positivity for serological markers of HBV infection and sociodemographic factors in a population-based study of 1243 adults aged 18–39 years, Arkhangelsk City, Russia, 2010–2011.

Variables	*n*	%	Prevalence (%) Serological Markers of HBV ^a^	Crude OR	95% CI ^b^
**Age**					
18–24 years	497	40.0	7.0	referent	
25–29 years	296	23.8	9.8	1.43	0.86–2.40
30–34 years	278	22.4	16.5	2.62	1.64–4.18
35–39 years	172	13.8	15.1	2.35	1.37–4.04
**Sex**					
Men	543	43.7	11.8	referent	
Women	700	56.3	10.3	0.86	0.60–1.23
**Education**					
Low (secondary education, 10–11 years or less)	209	16.8	15.8	referent	
Average (secondary vocational education)	302	24.3	9.9	0.59	0.35–0.999
High (incomplete higher and higher education)	732	58.9	10.0	0.59	0.38–0.92
**Marital status**					
Single	571	45.9	8.2	referent	
Widowed, divorced	86	6.9	14.0	1.81	0.92–3.57
Co-habitation	213	17.1	14.6	1.90	1.17–3.08
Married	373	30.0	12.3	1.57	1.02–2.41
**Income ^c^**					
Low income 1–4	207	16.7	15.5	referent	
Medium income 5–7	895	72.0	10.1	0.61	0.40–0.95
High income 8–10	141	11.3	9.9	0.60	0.31–1.18
**Total**	1243	100	10.9		

^a^ serological markers of HBV infection: HBsAg and/or anti-HBc; ^b^ significant association in bold; ^c^ subjective estimation by comparing own family income with family income on average in Arkhangelsk; Abbreviations: HBV: hepatitis B virus; OR: odds ratio; CI: confidence interval; HBsAg: HBV surface Antigen; anti-HBc: HBV core antibodies.

**Table 3:** The characteristic of the participants positive on HBs Ag (*N* = 15) in a population-based study of adults aged 18–39 years, Arkhangelsk City, Russia, 2010–2011.

Variables	*N* (%)
**Gender**	
Male	6 (40%)
Female	9 (60%)
**Median age**	30
**Ever being diagnosed with hepatitis B**	
No	13 (86.7%)
Yes	2 (13.3%)
**Injecting drug use**	
No	14 (93.3%)
Yes	0 (0%)
Do not want to answer	1 (6.7%)
Co-infection with hepatitis C virus *	
No	12 (80%)
Yes	3 (20%)
**Knowing someone with hepatitis B**	
No	8 (53.3%)
Yes	4 (26.7%)
Do not know	3 (20.0%)
**Ever being a blood donor**	
No	11 (73.3%)
Yes	4 (26.7%)
**Previous blood transfusions**	
No	15 (100%)
**Previous surgery**	
No	12 (80%)
Yes	3 (20%)
**Have you ever had sex with an intravenous drug user?**	
No	13 (86.7%)
Yes	1 (6.7%)
Do not know	1 (6.7%)
**Having tatoos**	
No	13 (86.7)
Yes	2 (13.3)

* positive on IgM + G.

**Table 4:** Prevalence of serological markers of HBV infection and crude associations between positivity for serological markers of HBV infection and behavioral factors in a population-based study of 1243 adults aged 18–39 years, Arkhangelsk City, Russia, 2010–2011.

Variables	*N*	%	Prevalence (%) Serological Markers of HBV ^a^	Crude OR	95% CI ^b^
Smoking					
Never smoked	491	39.5	8.6	referent	
Ever smoked	752	60.5	12.5	1.53	1.04–2.24
Binge drinking ^c^					
Never	242	19.5	9.1	referent	
Ever	1001	80.5	11.4	1.29	0.80–2.08
Self-reported STI (without HSV-2 or HIV)					
Never and don’t know	996	80.1	9.5	referent	
Yes	247	19.9	16.6	1.89	1.27–2.81
HSV-2 lab test					
Negative	1008	81.2	10.3	referent	
Positive	234	18.8	13.7	1.38	0.90–2.11
N of sexual partners in last 6 months					
0 and 1	941	75.7	9.9	referent	
≥2	302	24.3	14.2	1.51	1.03–2.23
Lifetime N of sexual partners					
0 and 1	134	10.8	6.7	referent	
2–5	424	34.1	9.9	1.53	0.72–3.23
6 and more	685	55.1	12.4	1.97	0.96–4.02
Use of condom during last sexual encounter					
Yes ^d^	339	27.3	8.6	referent	
No and don’t remember ^e^	904	72.7	11.8	1.44	0.93–2.21
Use of condom with a steady partner					
Always ^d^	270	21.7	8.9	referent	
Rarely and sometimes	563	45.3	10.7	1.22	0.74–2.01
Never	410	33.0	12.7	1.49	0.89–2.48
Use of condom with casual partner					
Always or never had casual partners	1112	89.5	11.0	referent	
Sometimes, rarely, never	131	10.5	10.7	0.97	0.54–1.74
Using of injected drugs					
Never	1209	97.3	10.8	referent	
Ever	34	2.7	14.7	1.42	0.54–3.73
Have you ever had sex with an intravenous drug user?					
No	1094	88.0	10.3	referent	
Yes	45	3.6	15.6	1.60	0.70–3.67
Don’t know	104	8.4	15.4	1.58	0.90–2.78
Age at sexual debut					
≥18 ^d^	517	41.6	9.5	referent	
≤17	726	58.4	12.0	1.30	0.90–1.88
Ever having sex with a partner of the same gender					
No	1197	96.3	10.9	referent	
Yes	46	3.7	13.0	1.23	0.51–2.96
Previous blood transfusions, surgery, ever being blood donor					
No medical procedures	551	44.3	10.9	referent	
One of these medical procedures	546	43.9	10.3	0.94	0.64–1.38
Two of these medical procedures	128	10.3	13.3	1.25	0.70–2.23
Three of these medical procedures	18	1.4	16.7	1.64	0.46–5.82
Having tattoos					
No	1071	86.2	10.6	referent	
Yes	172	13.8	13.4	1.31	0.81–2.12
Knowing someone with HBV infection					
No	865	69.6	10.2	referent	
Yes	184	14.8	14.7	1.52	0.96–2.42
Don’t know	194	15.6	10.8	1.07	0.65–1.77
Vaccination against HBV					
Yes, three doses	384	30.9	8.1	referent	
Yes, one or two doses	144	11.6	10.4	1.32	0.69–2.53
Don’t know	380	30.6	11.1	1.42	0.87–2.30
No	335	27.0	14.3	1.90	1.18–3.07
“Vaccination against HBV is necessary”					
Agree and tend to agree	1140	91.7	10.7	referent	
Disagree and tend to disagree	44	3.5	15.9	1.58	0.69–3.62
Don’t know	59	4.7	11.9	1.12	0.50–2.53

^a^ = serological markers of HBV infection: HBsAg and/or anti-HBc. ^b^ = significant association in bold. ^c^ = drinking 6 or more units in one occasion. ^d^: Includes 18 participants reporting no sexual debut. ^e^: Includes 11 participants who did not remember. Abbreviations: HBV: hepatitis B virus; OR: odds ratio; CI: confidence interval; STI: sexually transmitted infection; *N*: number; herpes simplex virus type 2 (HSV-2), HBsAg: HBV surface Antigen; anti-HBc: HBV core antibodies.

**Table 5:** Serological markers of HBV infection ^a^ and correlates by multivariable logistic regression analysis in a population-based study of 1243 adults aged 18–39 years, Arkhangelsk City, Russia, 2010–2011.

Variables	Adjusted OR	95% CI ^b^
Age		
18–24 years	Referent	
25–29 years	1.10	0.63–1.94
30–34 years	**1.99**	**1.15–3.43**
35–39 years	1.68	0.89–3.16
Education		
Low (secondary education (10–11 years) or less)	Referent	
Average (secondary vocational education)	0.61	0.35–1.06
High (incomplete higher and higher education)	0.75	0.46–1.22
Marital status		
Single	Referent	
Widowed, divorced	1.05	0.50–2.23
Co-habitation	1.73	1.00–2.98
Married	1.32	0.79–2.18
Income ^c^		
Low income 1–4	Referent	
Medium income 5–7	0.69	0.44–1.09
High income 8–10	0.70	0.35–1.41
Smoking		
Never smoked	Referent	
Ever smoked	1.25	0.81–1.91
Vaccination against HBV		
Yes, three doses	Referent	
Yes, one or two doses	1.36	0.70–2.66
Don’t know	1.40	0.84–2.33
No	**1.68**	**1.02–2.77**
Self-reported STI (without HSV-2 or HIV)		
Never and don’t know	Referent	
Yes	1.53	0.99–2.34
HSV-2		
Negative	Referent	
Positive	0.95	0.60–1.50
No. of sexual partners in the last 6 months		
0 and 1	Referent	
≥2	1.61	**1.02–2.54**
Lifetime no. of sexual partners		
0 and 1	Referent	
2–5	1.32	0.61–2.86
6 and more	1.14	0.52–2.49
Use of condom during last sexual encounter		
Yes ^d^	Referent	
No and don’t remember ^e^	1.20	0.67–2.15
Use of condom with a steady partner		
Always ^d^	Referent	
Rarely and sometimes	0.93	0.49–1.75
Never	0.93	0.46–1.87
Have you ever had sex with IDU?		
No	Referent	
Yes	1.16	0.48–2.81
Don’t know	1.18	0.64–2.19
Knowing someone with HBV infection		
No	Referent	
Yes	1.48	0.91–2.41
Don’t know	0.99	0.59–1.68

^a^ Serological markers of HBV infection: HBsAg and/or anti-HBc; ^b^ Significant association in bold; ^c^ Subjective estimation by comparing own family income with family income on average in Arkhangelsk; ^d^ Includes 18 participants reporting no sexual debut; ^e^ Includes 11 participants who did not remember; Abbreviations: HBV: hepatitis B virus; OR: odds ratio; CI: confidence interval; STI: sexually transmitted infection; herpes simplex virus type 2 (HSV-2), HBsAg: HBV surface Antigen; anti-HBc: HBV core antibodies; IDU: intravenous drug user. Significant results were marked in bold.
